# Dependence of Solidification for Bi_2_Te_3−x_Se_x_ Alloys on Their Liquid States

**DOI:** 10.1038/s41598-017-02507-4

**Published:** 2017-05-26

**Authors:** Yuan Yu, Zhan Wu, Oana Cojocaru-Mirédin, Bin Zhu, Xiao-Yu Wang, Na Gao, Zhong-Yue Huang, Fang-Qiu Zu

**Affiliations:** 1 Liquid/Solid Metal Processing Institute, School of Materials Science & Engineering, Hefei University of Technology, Tunxi Road 193, Hefei, 230009 China; 2 I. Physikalisches Institut (IA), Sommerfeldstraße 14, RWTH Aachen, 52074 Aachen Germany

## Abstract

The resistivity *versus* temperature (ρ-T) behaviours of liquid n-type Bi_2_Te_3−*x*_Se_*x*_ (x = 0.3, 0.45 and 0.6) alloys are explored up to 1050 °C. A clear hump is observed on all ρ-T curves of the three studied Bi_2_Te_3−*x*_Se_*x*_ melts during the heating process, which suggests that a temperature-induced liquid-liquid structural transition takes place in the melts. Based on this information, the solidification behaviours and microstructures of the alloys with different liquid states are investigated. The samples that experienced liquid structural transition show that the nucleation and growth undercooling degrees are conspicuously enlarged and the solidification time is shortened. As a result, the solidified lamellae are refined and homogenized, the prevalence of low-angle grain boundaries between these lamellae is increased, and the Vicker Hardness is enhanced. Atom probe tomography analyses prove that there is no segregation or nanoprecipitation within the grains, but the Te-rich eutectic structure and the evolution of composition near the Te-matrix phase boundary are investigated in a sample that experienced liquid structural transition. Our work implies that the solidification behaviours of Bi_2_Te_3−x_Se_x_ alloys are strongly related to their parent liquid states, providing an alternative approach to tailor the thermoelectric and mechanical properties even when only a simple solidification process is performed.

## Introduction

With the increasing concern of environmental pollution and energy crisis, thermoelectric (TE) materials, which can directly interconvert electrical energy and thermal energy, have received considerable attentions^1, 2^. Bi_2_Te_3_-based solid solutions, including (Bi, Sb)_2_Te_3_ and Bi_2_(Te, Se)_3_, are known as the best thermoelectric materials at temperatures from 300 K to 500 K^3, 4^. In addition, these alloys also exhibit outstanding properties as phase change materials^5^ and topological insulators^6^. The commercial Bi_2_Te_3_-based TE legs are generally prepared by unidirectional solidification methods, such as zone melting^7^, Bridgman^8^, and Czochralski^9^. Peak TE figure of merit values of these melt-grown ingots are typically in the range of 0.8 to 1.1 along the preferentially oriented ab-plane^4, 10^. However, the lamellar structure and weak van der Waals bond between the Te-Te layers make them susceptible to cleavage along this basal plane, imparting very poor mechanical properties, and hence limiting their application potentials^10^. To improve the TE performance and mechanical properties of Bi_2_Te_3_-based compounds, various alternative processing techniques have been widely implemented, such as ball milling or melt spinning plus powder sintering^11, 12^, hot deformation^13^, and high-pressure fabrication^14^. Although these methods have been shown efficient to improve the thermoelectric properties, the relatively high energy consumption, low production efficiency, and relatively sophisticated equipment required for production processes cannot be overlooked. Therefore, it is worthwhile to attempt to synthesize bulk TE materials with satisfactory microstructures and properties via a free solidification method.

There has been a notable trend in engineering practices that the properties and solidified microstructures of many alloys are dependent on the thermal history of their parent liquids^15–18^. Both theoretical and experimental results suggest that the liquid structures and their properties might be discontinuously changed as a function of pressure or temperature^19–21^. Thus, the alteration of the solidification microstructures due to different thermal histories may be ascribed to their different liquid states. Hitherto, the temperature-induced liquid-liquid structural transition (TI-LLST) has been suggested to occur in some single- and multiple-component melts, such as Bi-Te^15, 16^, Pb-Bi^22^, and Bi-Sb^23^ by various reports. More significantly, changing the liquid states, based on the TI-LLST behaviour, has been confirmed to be effective in regulating the solidification processes and microstructures^15–17, 22, 23^.

Our previous studies demonstrated the feasibility of changing the liquid state to modify the solidification microstructures and TE properties of p-type Bi_2_Te_3_-based alloys^15, 16^. This motivates us to further explore the effect of TI-LLST on their n-type counterparts. Hence, in this work, the resistivity-temperature (ρ-T) behaviours of the *n*-type Bi_2_Te_3−*x*_Se_*x*_ (x = 0.3, 0.45, 0.6) liquid alloys were explored, the results of which suggested the occurrence of an irreversible TI-LLST during the first heating process. Moreover, after experiencing TI-LLST, the microstructures of the Bi_2_Te_3−*x*_Se_*x*_ alloys were effectively refined compared with those obtained from the conventional solidification conditions, which leads to the increased amount of low-angle grain boundaries and enhanced Vicker Hardness. Atom probe tomography (APT) analysis was carried out, to our best knowledge, for the first time on this kind of semiconductor. The results show that the solidified microstructures are very homogenous at the nanoscale without segregation or precipitation in the grain interior. Nevertheless, the Te-rich eutectic structure was investigated in the sample which experienced TI-LLST. The composition evolution near the interface between the Te-rich phase and the matrix at nanometre scale was also analysed by APT. It is believed that the interrelationship between the parent liquid states and the solidified microstructures uncovered by this work may help to better understand the solidification process of this kind of material. Furthermore, it provides a new control over the structural configuration of TE materials with possible relevance to improvement of their properties.

## Methods

According to the stoichiometry of Bi_2_T_3−*x*_Se_*x*_ (x = 0.3, 0.45, 0.6) alloys, high purity (5 N) elemental granules of Bi, Te and Se were weighed and then melted at 650 °C for 3 hours under B_2_O_3_ flux protection. Subsequently, the melted samples were poured into quartz measuring cells to record the temperature-dependent liquid-state resistivity using the DC four-probe method in an electrical furnace at ramping and cooling speeds of 5 °C/min under high-purity Argon (5 N) atmosphere^16^. These samples were only used to investigate the liquid resistivity behaviours *versus* temperature. The same measurements were repeated three times, and the results were well reproducible.

To explore the relationship between solidification behaviour and different liquid states, six new samples were prepared. Smelting processes were carried out according to the anomalous humps on the ρ-T curves, as specified in Table 1. The smelting process at temperatures below the hump is a conventional one, which normally possesses an overheat degree of near 100 °C. Here, the high-temperature smelting process (at least 300 °C overheated) is designed to induce the potential TI-LLST to obtain distinct liquid states. In order to exclude the influence of solidification temperature field on the microstructures, all samples were maintained at 650 °C for 1 h after the smelting process and then air-cooled with crucibles and protective covers. The cooling processes were recorded utilizing a Keithley-2182 Nanovoltmeter with K-type thermocouples. Six cylindrical ingots were obtained after cooling. More experimental details can be found in our previous paper^16^.

**Table 1:** Specific sample smelting processes for Bi_2_T_3−*x*_Se_*x*_ (x = 0.3, 0.45, 0.6) alloys.

Sample	x = 0.3A^a^	x = 0.3 B^b^	x = 0.45 A	x = 0.45 B	x = 0.6 A	x = 0.6 B
Composition	Bi_2_Te_2.7_Se_0.3_		Bi_2_Te_2.55_Se_0.45_		Bi_2_Te_2.4_Se_0.6_	
Transition ranges	790.6~900.4 °C		810.1~940.5 °C		800.7~920.6 °C	
Smelting process	750 °C; 1.5 h	900 °C; 1.5 h	750 °C; 1.5 h	1000 °C; 1.5 h	750 °C; 1.5 h	950 °C; 1.5 h
T− P^c^ process	650 °C; 1 h (for all samples)					

These samples are used for investigating the solidification processes and corresponding microstructures and mechanical properties. ^a^A represents smelted at temperatures below the range of TI-LLST. ^b^B represents smelted at temperatures above the range of TI-LLST. ^c^T− P represents temperature-preservation.

**Figure 1:**
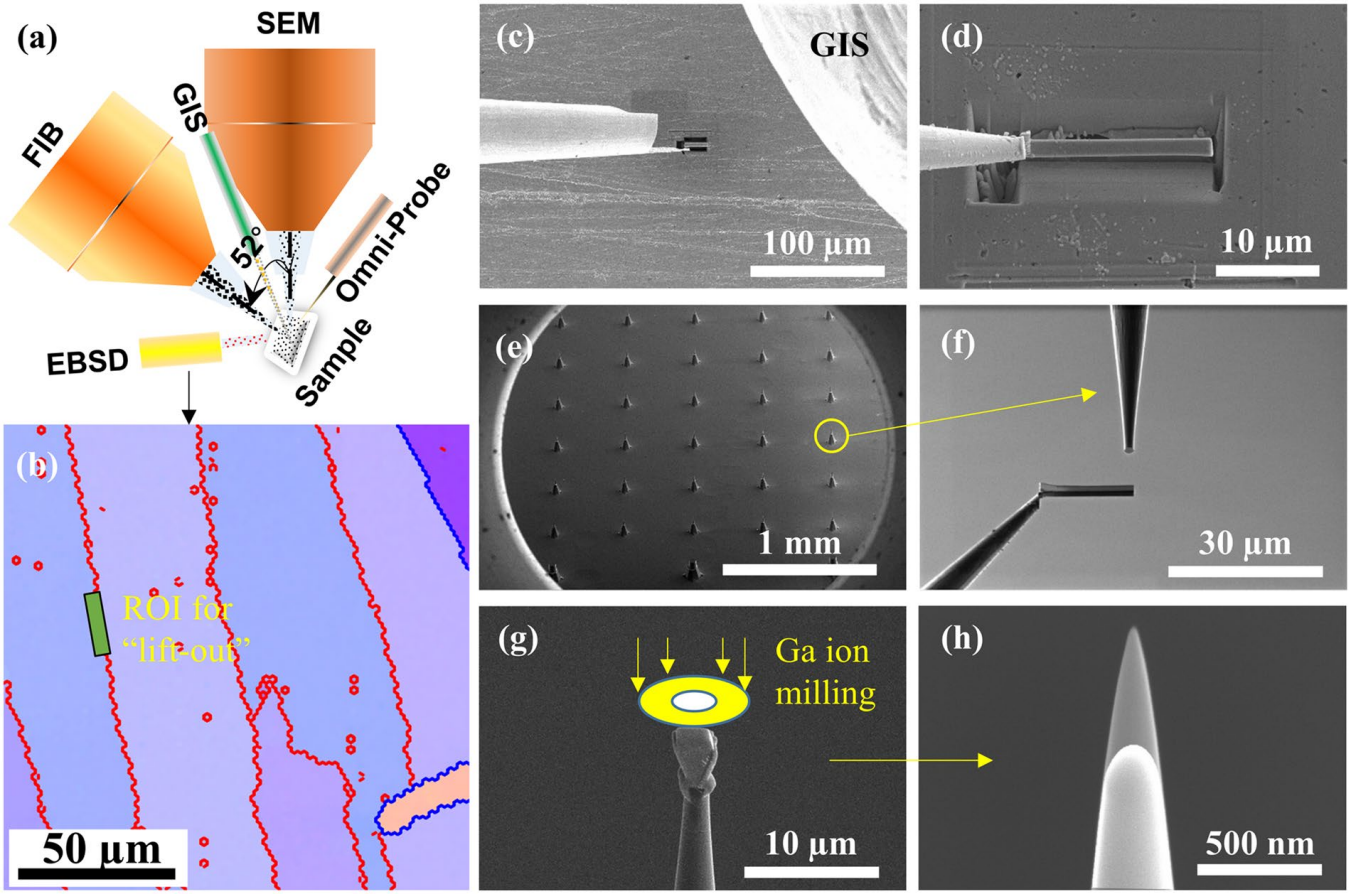
Site-specific “lift-out” process for preparing APT tips. (**a**) Schematic diagram of SEM-FIB dual beam system with EBSD attachment; (**b**) EBSD sample surface overlapped with grain boundaries. The green bar is the region of interest (ROI) for the “lift-out” process; (**c**) The micromanipulator (Omni-probe, left) and the gas injection system (GIS, right); (**d**) The Omni-probe and the ROI of sample are welded with Pt; (**e**) Microtip^TM^ array coupon with 36 Si post; (**f**) Enlarged view of Si post and the sample before mounting on it; (**g**) One tip before suffering the Ga ion annular milling; (**h**) The APT tip profiles before (longer) and after (shorter) the measurement with tip radius of near 50 nm and 130 nm, respectively.

Various techniques were utilized to characterize the structural and chemical properties of these as-cast ingots. The samples were polished and then etched in HCl-9% H_2_O_2_(*L*) for the optical microscopy observation. X-ray diffraction (XRD, D/MAX2500 V, Rigaku) was operated at 40 kV/40 mA using *Cu* − *k*_*α*_ radiation (*λ* = 1.5406 Å). Scanning electron microscopy (SEM) with energy dispersive spectrometer (EDS, Oxford) and electron back-scatter diffraction (EBSD, EDAX) were carried out on a SEM-FIB dual beam system (Helios 650, FEI). Atom probe tomography (APT) was utilized to investigate the three-dimensional (3D) elemental distribution at nanometre scale. The APT tips were prepared using the site-specific “lift-out” process in a SEM-FIB dual beam system (Helios 650) as schematically shown in Fig. 1. The APT tips were field evaporated in a local electrode atom probe (LEAP^®^ 4000 X Si, Cameca) by applying a picosecond ultraviolet (UV) laser (wavelength = 355 nm). The experimental parameters for APT are as follows: base temperature of 50 K, laser pulse energy of 30 pJ, pulse repetition rate of 200 kHz, detection rate of 1%, flight length of 160 mm. The detector efficiency is about 50% due to the morphology of the microchannel plate (MCP). The APT data was analysed using IVAS 3.6.12^TM^. The Vicker Hardness measurement was conducted on a MH-3L equipment with a load of 0.245 N and holding time of 10 s. Ten data points were recorded for each sample.

### Data Availability

The datasets generated during and/or analysed during the current study are available from the corresponding author on reasonable request.

## Results and Discussion

**Figure 2:**
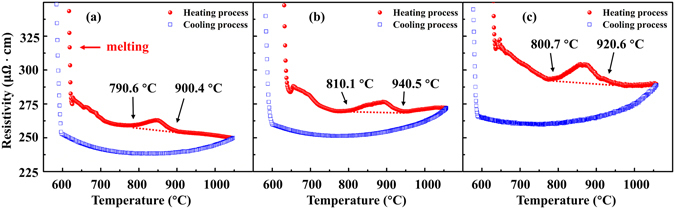
The *ρ*-*T* curves of liquid Bi_2_Te_3−*x*_Se_*x*_ alloys during heating and cooling processes (5 °C/min). (**a**) *x* = 0.3; (**b**) *x* = 0.45; (**c**) *x* = 0.6.

The plots of electrical resistivity (ρ) of Bi_2_Te_3−*x*_Se_*x*_ (x = 0.3, 0.45, 0.6) liquid alloys as a function of temperature during heating and cooling processes are shown in Fig. 2. During melting the electrical resistivity decreases abruptly, which can be attributed to the alloys’ semiconductor character^24^. After melting, the ρ-T curves of liquid alloys show a nonlinear relationship, which is different from the behaviour of metals^24^. As the temperature further increases, intriguingly, an anomalous hump arises on the ρ-T curves of liquid Bi_2_Te_2.7_Se_0.3_, Bi_2_Te_2.55_Se_0.45_, and Bi_2_Te_2.4_Se_0.6_ within 790.6–900.4 °C, 810.1–940.5 °C, and 800.7–920.6 °C during the heating process, respectively. However, the resistivity curves become smooth in the subsequent cooling processes, which suggests that the liquid state change is irreversible. The physical mechanism of this anomalous hump on the ρ-T curve was discussed in our previous work^15, 16, 25^. This abnormal phenomenon is well reproducible in several repeated experiments. The similar phenomena were also observed in pure Bi and Bi- and Sb-based melts and cross verified by DSC, DTA, internal friction, and XRD methods^22, 26, 27^. Since the electrical resistivity is structure sensitive according to the generalized Faber-Ziman theory^28^, the anomalous phenomena on the ρ-T curves allude to the occurrence of an irreversible TI-LLST in the Bi_2_Te_3−*x*_Se_*x*_ liquid alloys.

**Figure 3:**
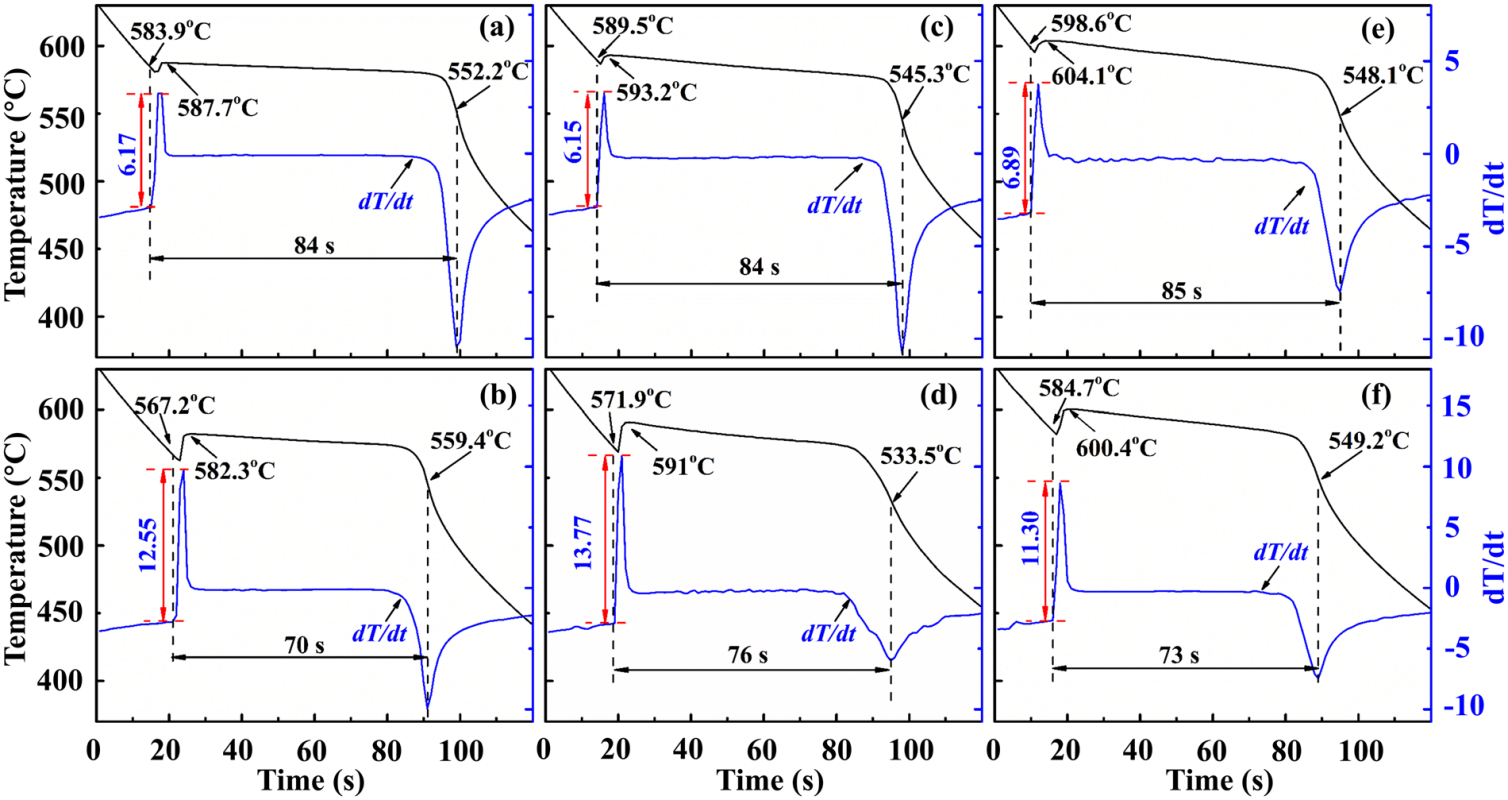
Cooling curves for Bi_2_Te_3−*x*_Se_*x*_ liquid alloys. The temperature indicated by the first arrow from the left side of each picture is the nucleation temperature, *T*_*N*_. The second arrow indicates the recalescence temperature, *T*_*G*_. The red double sided arrow shows the height of the first peak for *dT*/*dt* curve, *Δh*. The black double sided arrow shows the solidification time. (**a**) x = 0.3-A; (**b**) x = 0.3-B; (**c**) x = 0.45-A; (**d**) x = 0.45-B; (**e**) x = 0.6-A; (**f**) x = 0.6-B.

According to the resistivity anomaly temperatures, the smelting and solidification experiments on Bi_2_Te_3−*x*_Se_*x*_ alloys in different liquid states were carried out as described in the experimental part. The cooling curves of all samples were recorded for exploring the solidification behaviour during the solidification process. The characteristic solidification parameters were calculated by the Newton thermal analysis method^29^. Figure 3 shows that the cooling curve mainly evolves along four steps. The fast decline, in the beginning, corresponds to the liquid cooling process with a near constant cooling rate as implied by the first plateau of the first-order differentiation (*dT*/*dt*) curve. After that, the liquid alloy starts to largely nucleate and release the solidification latent heat, leading to the abrupt ascent of the *dT*/*dt* curve. The maximum value of *dT*/*dt* is related to the recalescence temperature, which is also the crystal growth temperature (*T*_*G*_). During the crystal growth process, both liquid and solid states coexist. Finally, when all liquid phases transform into solid states, the solidified ingot cools down very fast (abrupt decline of the *dT*/*dt* curve) in the ambient condition. The calculated results from the cooling curves, as shown in Fig. 3 and Supplementary Table S1, demonstrate that the characteristic solidification parameters for alloys solidified from distinct liquid states are quite different. Obviously, the nucleation temperature (*T*_*N*_) of the B-group specimens (with TI-LLST) is significantly lower than that of the A-group specimens (without TI-LLST), which means that the solidification undercooling degree for B-group specimens is larger. The height of the first peak (*Δh*) for *dT*/*dt* curve of the B-group specimens is markedly larger than that of the A-group specimens, which is believed to arise from the larger nucleation rate. During the crystal growth stage, the *T*_*G*_ of B-group specimens is lower than that of their comparative counterparts, and the crystal growth time of the B-group specimens is also significantly shortened. The decreased crystal growth time, on the one hand, comes from the larger nucleation rate and more homogeneously distributed nuclei, and on the other hand, is on account of the lower growth temperature which provides larger growth driving force^30^. In the following context, we offer an explanation of the observation that the two sets of samples present distinguishable solidification behaviours and microstructures.

As is well known, the liquid structures at temperatures not far above liquidus are normally inhomogeneous, containing solid-like, topologically short-range ordered and/or chemically short-range ordered clusters^20^. These clusters dissipate and engender new ones, but they do not vanish; they have statistical structures, sizes, and constituents that change continuously with temperature. Additionally, it is more widely accepted that polymorphism exists in liquid states^31^, and critical points between stable liquids or/and metastable ones are revealed^32, 33^. Mudry^34^ reported that, not far from the liquidus, the structure of liquid Bi-Te alloys in the range of 52–60 at.% Te still possesses short-range ordered clusters that are similar to the solid structure of Bi_2_Te_3_. Neutron diffraction studies also indicate that the crystalline Bi_2_Se_3_-like configuration of Bi-Se pairs remains to some extent in the liquid state^35, 36^. The interatomic bonds in original clusters will be broken when the temperature reaches the critical scope. During this process, the original clusters are reduced, and new clusters with smaller size are formed. These changes impact solidification by inducing a much smaller critical nucleation radius, r*^30^. In addition, since the solid-like clusters are destroyed during the TI-LLST process, the structural mismatch at the solid-liquid interface is exaggerated. Therefore, the solid-liquid interface energy (*σ*_*SL*_) should be higher for B-group samples. According to the classical nucleation theory^37^, the undercooling degree for nucleation, Δ*T*, can be expressed as:

1 $${\rm{\Delta }}T=\frac{2{\sigma }_{SL}{V}_{S}{T}_{m}}{{\rm{\Delta }}{H}_{m}{r}^{\ast }}$$

where *V*_*S*_ is the molar volume of nucleation crystal, *T*_*m*_ is the melting point, and Δ*H*_*m*_ is the latent heat of fusion. Therefore, the Δ*T* will rise with *σ*_*SL*_ increasing and r* decreasing, which is in good agreement with the lower nucleation temperature in B-group samples. The larger undercooling degree, higher nucleation rate, and higher fraction of nucleation sites will contribute to much finer solidification structures^30, 37^.

**Figure 4:**
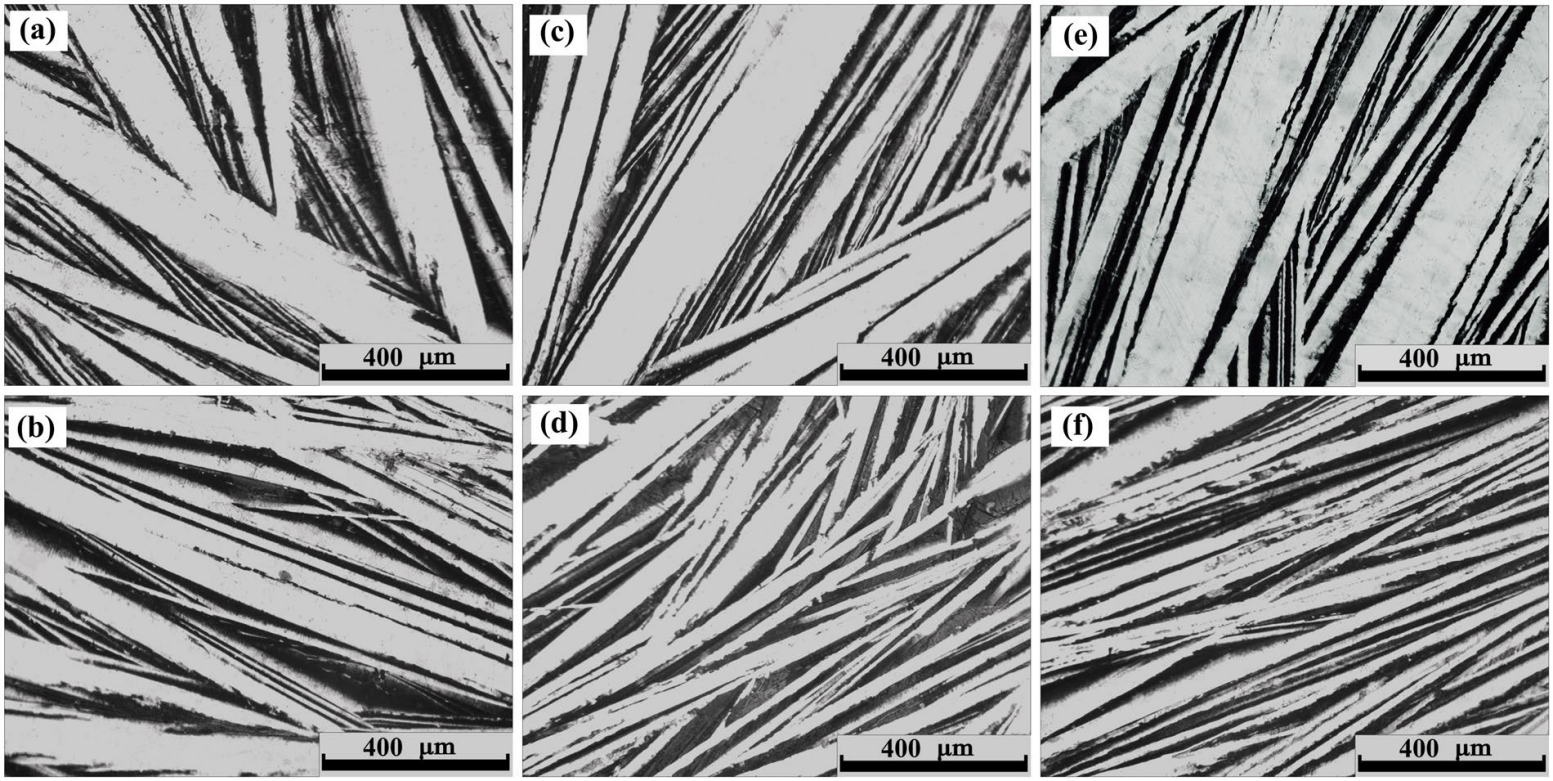
Solidification microstructures as observed by optical microscopy along the radial direction of the cylindrical ingot for both A-group (upper row) and B-group (lower row) samples. (**a**) Bi_2_Te_2.7_Se_0.3_-A; (**b**) Bi_2_Te_2.7_Se_0.3_-B; (**c**) Bi_2_Te_2.55_Se_0.45_-A; (**d**) Bi_2_Te_2.55_Se_0.45_-B; (**e**) Bi_2_Te_2.4_Se_0.6_-A; (**f**) Bi_2_Te_2.4_Se_0.6_-B.

As indicated by the cooling curves, the microstructures are expected to be refined in B-group samples. Therefore, optical microscopy, SEM, EBSD, and APT were utilized here to investigate the structural divergence for ingots solidified out of the liquid with different states (without and with experiencing TI-LLST). Figure 4 shows that the microstructures are mainly composed of alternative lamellae. The microstructures become finer and more homogenized in morphology for all B-group samples.

**Figure 5:**
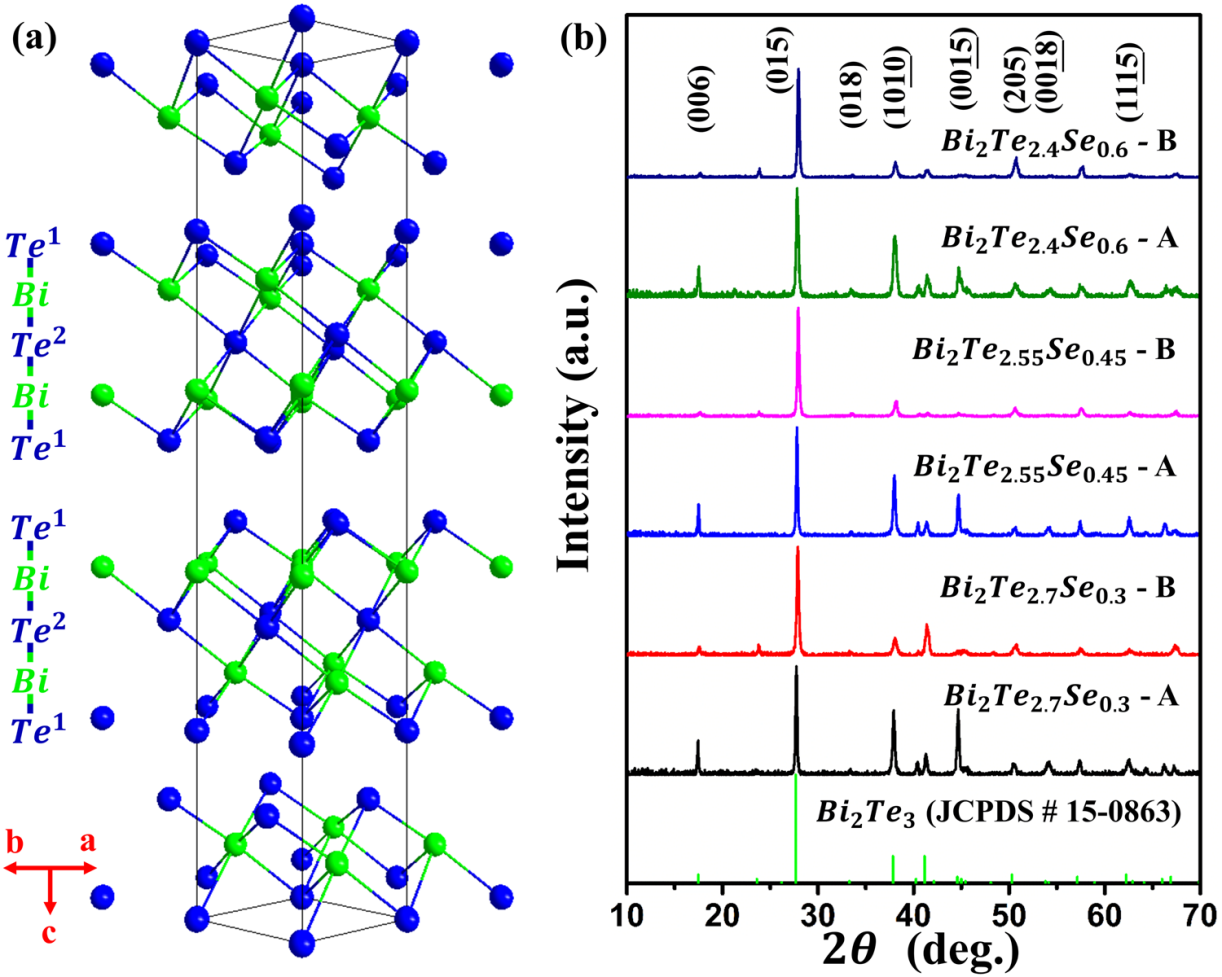
(**a**) Schematic diagram of the Bi_2_Te_3_ crystal structure; (**b**) XRD pattern of radial surfaces of as-cast Bi_2_T_3−*x*_Se_*x*_ (x = 0.3, 0.45, 0.6) samples solidified from different liquid states.

**Figure 6:**
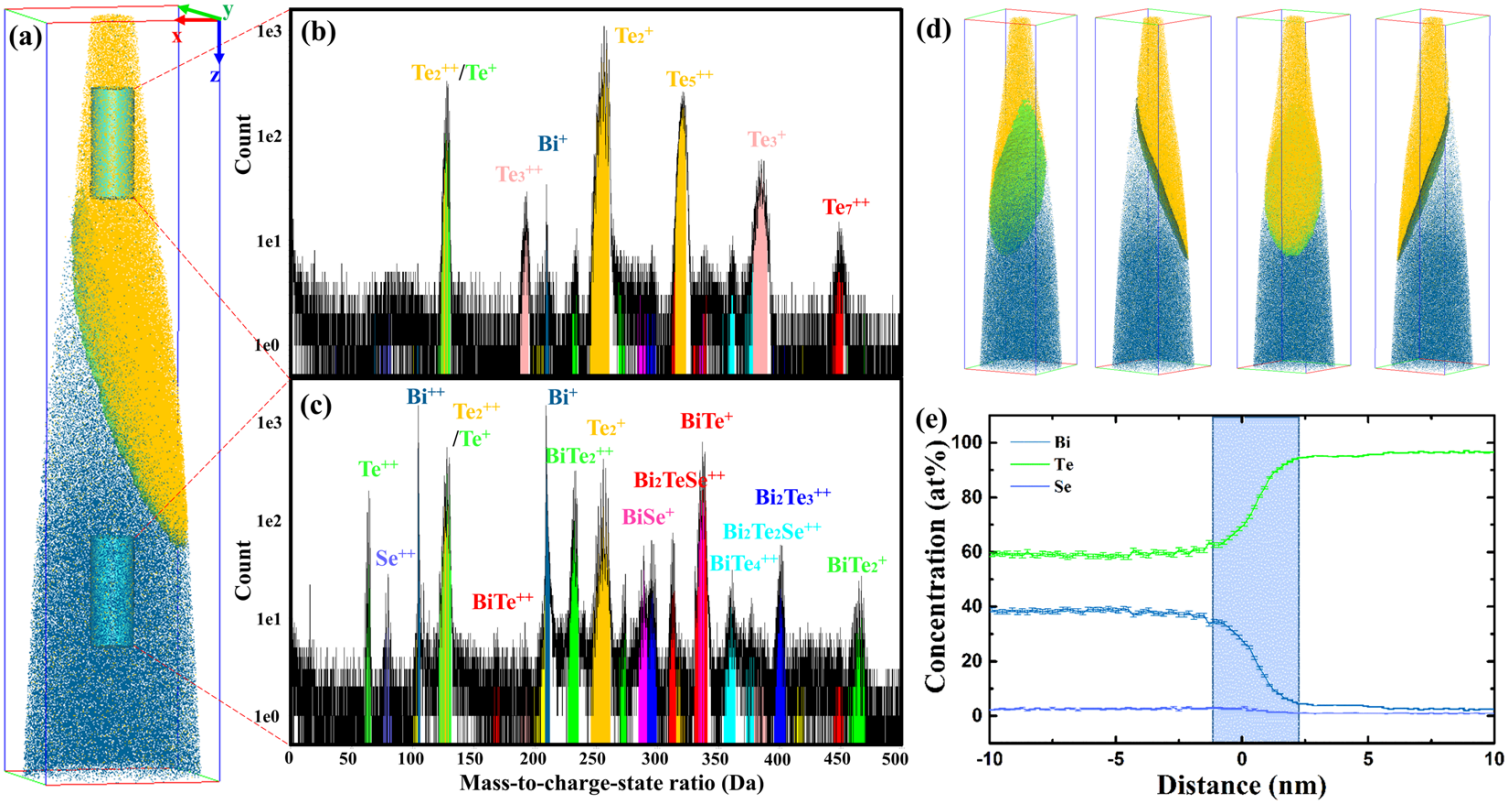
APT results of sample Bi_2_Te_2.55_Se_0.45_-B. (**a**) Three-dimensional elemental maps showing the interface between a main-phase grain and a Te-rich grain (for clarity, only Te_5_ and Bi ions are shown). The analysed volume is 80 × 80 × 350 *nm* ^3^; (**b**) mass spectrum of the cylindrical ROI from the upper part of the tip; (**c**) mass spectrum of the cylindrical ROI from the lower part of the tip; (**d**) different view directions showing the iso-concentration surface of 80 at% Te; (**e**) Proximity histogram (Proxigram) of the iso-surface of 80 at% Te. The shadow area depicts the diffusive area at the interface between the Te-rich phase and the matrix.

In order to characterize the possible phases and their compositions in the samples, XRD and EDS analysis were carried out on the surface parallel to the radial direction of solidified ingots. All XRD patterns (Fig. 5(b)) can be matched with the standard pattern of Bi_2_Te_3_ (JCPDS 15–0863, rhombohedral, ${\rm{R}}\bar{3}m$, as shown in Fig. 5(a)) with only small deviations of the peak positions, which indicates that both phases, the bright and dark regions in Fig. 4, are solid solutions of Bi_2_Te_3_. The EDS measurements were carried out to investigate the chemical composition of all six samples, shown in the Supplementary Figure S1 and Table S2. The results imply that the concentration between adjacent lamellae is slightly different. However, both XRD and EDS have a limited chemical and spatial resolution due to the penetration area, typically from few hundred nm to few µm. Therefore, APT analysis was performed to investigate the chemical and structural homogeneity in three dimensional space. APT is a destructive characterization technique based on the field evaporation theory. It is particularly effective at chemically and structurally characterizing materials in 3D at the nanometer scale^38, 39^. We analysed two samples, Bi_2_Te_2.55_Se_0.45_-A and Bi_2_Te_2.55_Se_0.45_-B. Six APT tips were measured for each sample. Since the grain sizes of the measured samples are at the micrometre scale, while the APT tips are at the nanometre scale, it is rather difficult to get the grain boundary within the APT tips. Hence, only the single grain was analysed for sample Bi_2_Te_2.55_Se_0.45_-A, and the composition is very homogeneous in the grain interior (the results can be found in the Supplementary Information, Figure S2). Intriguingly, a very Te-rich phase was found in sample Bi_2_Te_2.55_Se_0.45_-B. According to the binary phase diagram of Bi-Te alloy^10^, the Te-rich phase is the eutectic phase. Figure 6(a) shows the 3D reconstruction of the volume analysed by APT. Mass spectra of the cylindrical regions of interest (ROIs) are shown in Fig. 6(b) and (c). The mass spectrum of the upper ROI shows that the peaks are mainly formed by Te and its isotopes, while the mass spectrum of the lower ROI shows very complex peaks formed by Bi, Te, Se, and their combinations. In order to analyse the composition evolution near the interface, an iso-concentration surface of 80 at% Te is created as highlighted by the green “leaf-like” plane. Figure 6(d) displays its shape from different viewing directions. The proximity histogram (proxigram), Fig. 6(e), is calculated based on the iso-surface of 80 at% Te. The results of the proxigram clearly present the very Te-rich phase, which contains approximately 97 at% Te. The interface between the Te-rich phase and the matrix has a diffusive region with big variations of Te and Bi concentrations within ~3 nm, as highlighted by the shadow area in Fig. 6(e). This variation of composition will influence the local structural environment and carrier and phonon transport processes. No compositional fluctuation or precipitation was found in the interior of the matrix grain of sample Bi_2_Te_2.55_Se_0.45_-B, consistent with the analysis of sample Bi_2_Te_2.55_Se_0.45_-A. Since APT is a nanoscale characterization technique, we cannot give the conclusion that there is no Te-rich phase in sample Bi_2_Te_2.55_Se_0.45_-A. However, from the viewpoint of solidification, we may conjecture that the Te-rich phase is more prone to form in B-group samples. The partition coefficient is defined as ${k}_{0}={C}_{S}^{\ast }/{C}_{L}^{\ast }$, where ${C}_{S}^{\ast }$ and ${C}_{L}^{\ast }$ are the concentrations of solute in the solid phase and liquid phase, respectively, at a specific solidification temperature. Given a nominal solute concentration C_0_, in the beginning of solidification, the concentration of solute in the solid phase is *C*_0_ *k*_0_. When *k*_0_ < 1, the extra solute will be repelled out from the solid phase. Thus, the solute concentration in the liquid phase not far from the solid-liquid interface will increase and then reach *C*_0_/*k*_0_. The smaller *k*_0_ (for *k*_*0*_ < 1) value, the larger difference of solute concentration at solid-liquid interface. The solidification partition coefficient (*k*_*0*_) for Te in Bi-Te system is less than unity (*k*_*0*_ < 1), thus Te will be rejected by the solid at the solid-liquid interface during the solidification process^40^. Moreover, it is reported that the effective partition coefficient is decreased after experiencing TI-LLST^41^. The decrease of the partition coefficient leads to more Te being repelled from the solid-liquid interface. In addition, the pre-solidified matrix structures will form grooves between them, where the rejected Te is very easy to aggregate. With the enrichment of Te and decreasing of temperature, the eutectic reaction occurs at Te concentration of near 90 at% in the grooves. The eutectic structure is composed of pure Te and some small structures that have compositions similar to that of the matrix. APT data shows the composition of the eutectic Te is 97 at% because of the mutual diffusion between Te and Bi. The samples experienced TI-LLST have a higher nucleation rate and larger fraction of nuclei, leading to more solid-liquid interface. The eutectic reaction occurs at the Te-rich regions near the solid-liquid interface. Therefore, the increased solid-liquid interfaces increase the volume fraction of the Te-rich phase.

**Figure 7:**
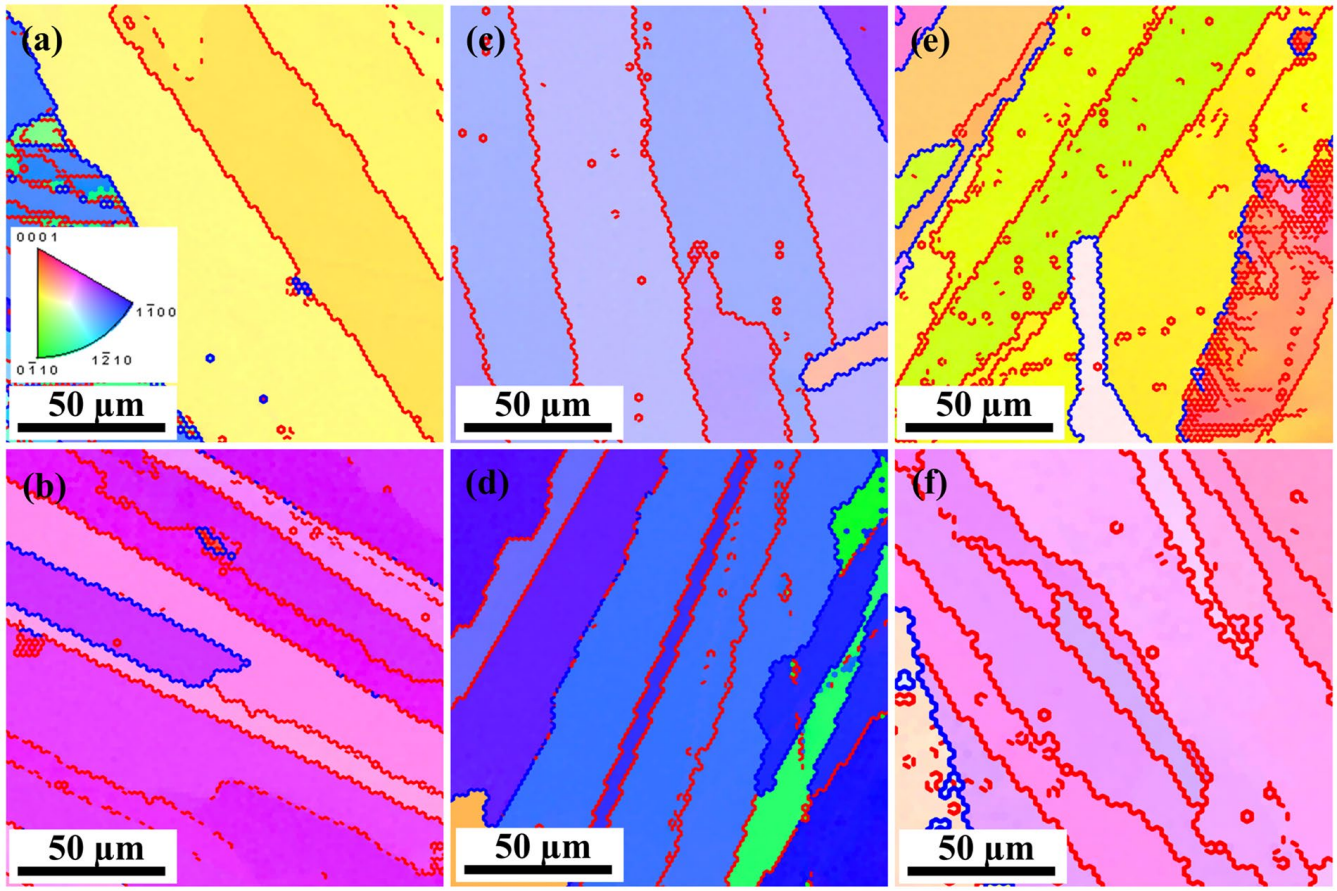
EBSD inverse pole figure maps covered with misorientation lines for grain boundaries. The red and blue lines represent the misorientation angles between 2°~15° (LAGB) and 15°~180° (high-angle GB), respectively. All the panels share the same legend of orientation map. (**a**) Bi_2_Te_2.7_Se_0.3_-A; (**b**) Bi_2_Te_2.7_Se_0.3_-B; (**c**) Bi_2_Te_2.55_Se_0.45_-A; (**d**) Bi_2_Te_2.55_Se_0.45_-B; (**e**) Bi_2_Te_2.4_Se_0.6_-A; (**f**) Bi_2_Te_2.4_Se_0.6_-B.

EBSD measurements were carried out to investigate the orientation relationship between two adjacent lamellae. As shown in Fig. 7, the misorientation angle between two adjacent lamellae is very often with values smaller than 15°, referring thus to low-angle grain boundaries (LAGBs). Normally, the LAGBs are composed of an array of dislocations^42^. These dislocations generate a strain field nearby, leading thus to a more pronounced phonon scattering at grain boundaries^43^. Up to now, one of the highest thermoelectric figure-of-merit values of bulk Bi_2_Te_3_-based material has been achieved by embedding dense dislocation arrays in grain boundaries^44^. The (0001) pole figures also verify the strong preference for similar orientation of adjacent lamellae for all 6 samples, Supplementary Figure S3. It is found that electrons transport more easily in such textured structures, while the Seebeck coefficient is less influenced by the crystal orientation, and such structures are thus beneficial for higher electrical conductivity and thermoelectric power factor^45^. Additionally, the small deviation in the composition of contiguous lamellae translates into a small degree of mass fluctuation at the boundary. This mass fluctuation can contribute to scattering of the heat-carrying phonons and may also serve as an energy barrier to filter out the low-energy carriers^46, 47^. Thus, a high thermoelectric performance can be expected if these specular structures are rationally tailored, and the distinct solidification behaviours and microstructures from different liquid states supply an additional control over the structural modification.

**Figure 8:**
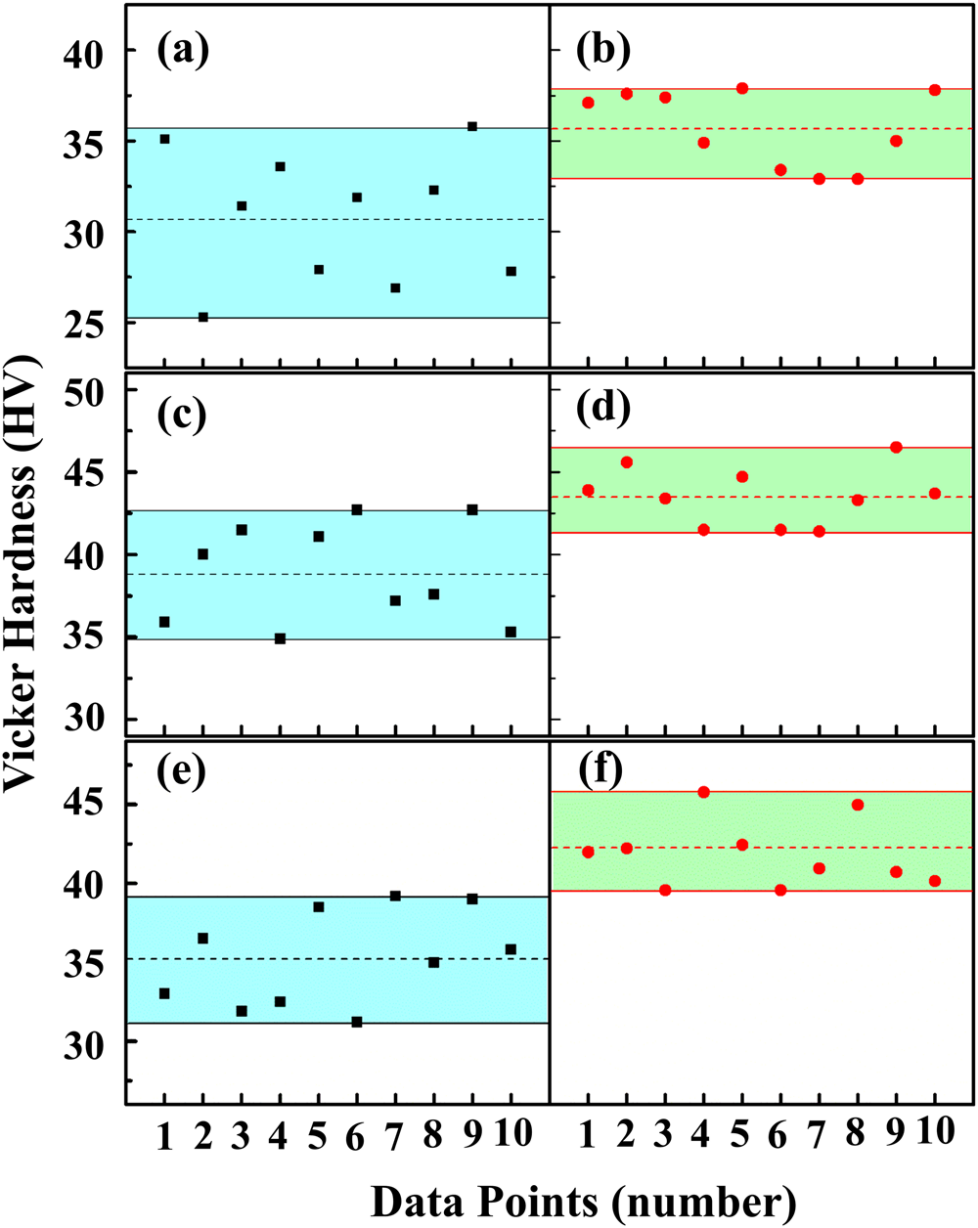
Vicker Hardness of all samples. The loading force and loading time are 0.245 N and 10 s, respectively. (**a**) Bi_2_Te_2.7_Se_0.3_-A; (**b**) Bi_2_Te_2.7_Se_0.3_-B; (**c**) Bi_2_Te_2.55_Se_0.45_-A; (**d**) Bi_2_Te_2.55_Se_0.45_-B; (**e**) Bi_2_Te_2.4_Se_0.6_-A; (**f**) Bi_2_Te_2.4_Se_0.6_-B.

Owing to the weak van der Waals force between Te^(1)^ −Te^(1)^ layers, Fig. 5(a), the highly textured samples are vulnerable to fracture along this plane, largely restricting the working condition and service time of the thermoelectric devices, as well as wasting raw materials in cutting and other machining operations^3^. Therefore, more and more attention has been paid to improve the mechanical properties of Bi_2_Te_3_-based materials in recent studies^48–50^. As one example of TI-LLST influence on mechanical properties, the hardness was measured for all as-cast samples. Figure 8 demonstrates that the Vicker Hardness is enhanced and varies less for all B-group samples, which comes from the refined and homogenized microstructures solidified from the melt after experiencing TI-LLST. As with the less simple powder metallurgy and mechanical deformation methods^48–50^, our free-solidification method with liquid state manipulation shows an ability to enhance the mechanical performance.

## Conclusions

The abnormal humps on the ρ-T curves suggest that an irreversible TI-LLST occurs during the first heating process for all Bi_2_Te_2.7_Se_0.3_, Bi_2_Te_2.55_Se_0.45_, and Bi_2_Te_2.4_Se_0.6_ liquid alloys in temperature ranges of 790.6–900.4 °C, 810.1–940.5 °C, and 800.7–920.6 °C, respectively. It is assumed that the structural transition results from the dissolution of short-range ordered Bi_2_Te_3_, Bi_2_Se_3_, and Bi-Te-Se ternary clusters. Based on this information, the samples solidified out of the liquids with TI-LLST exhibit significantly different solidification behaviours: the solidification undercooling degree is increased; the nucleation rate is enhanced; the crystal growth time is shortened. These changes lead to refinement of solidification microstructures, an increased prevalence of low-angle grain boundaries, and enhancement of Vicker Hardness. The Te-rich eutectic phase was observed by the state-of-the-art APT technique. The composition evolution near the matrix-Te phase boundary at the nanometre scale also was analysed, which may help to better understand the carrier transport process at the interface. It is reasonable to conjecture that these structural alterations might lead to an enhancement of the thermoelectric and mechanical properties of commercial-scale ingots, thus benefitting applications.

## Electronic supplementary material

Supplementary Information
