# Supplementary material for: Dependence of Solidification for Bi2Te3−xSex Alloys on Their Liquid States
Source: Sci Rep. 2017 May 26;7:2463. doi: 10.1038/s41598-017-02507-4 (PMC5446403; doi:10.1038/s41598-017-02507-4)
Supplement: Supplementary file 1 — Supplementary Information [file 41598_2017_2507_MOESM1_ESM.pdf]

Supplementary Information

Dependence of Solidification for $Bi_2Te_{3-x}Se_x$ Alloys on Their Liquid States

Yuan Yu,[ab] Zhan Wu,[a] Oana Cojocaru-Mirédin,[b] Bin Zhu,[a] Xiao-Yu Wang,[a] Na Gao,[a] Zhong-Yue Huang,[a]** and Fang-Qiu Zu[a]*

[a] Liquid/Solid Metal Processing Institute, School of Materials Science & Engineering, Hefei University of Technology, Hefei 230009, China

[b] I. Physikalisches Institut (IA), Sommerfeldstraße 14, RWTH Aachen, 52074, Aachen, Germany

* corresponding author: fangqiuzu@hotmail.com

** corresponding author: zyhuang1981@hotmail.com

Contents:

Figure S1. SEM pictures and marked points where the EDS measurements were carried out.

Figure S2. Atom probe tomography results of sample $Bi_2Te_{2.55}Se_{0.45}$-A;

Figure S3. Pole figures of (0001) plane generated by the EBSD resutls as shown in Fig. 7 in the main text.

Table S1. Characteristic temperatures and parameters in cooling curves of $Bi_2Te_{3-x}Se_x$ melts;

Table S2. Atomic composition ratios of Bi, Te, and Se obtained from the EDS spectra as shown in **Figure S1**.

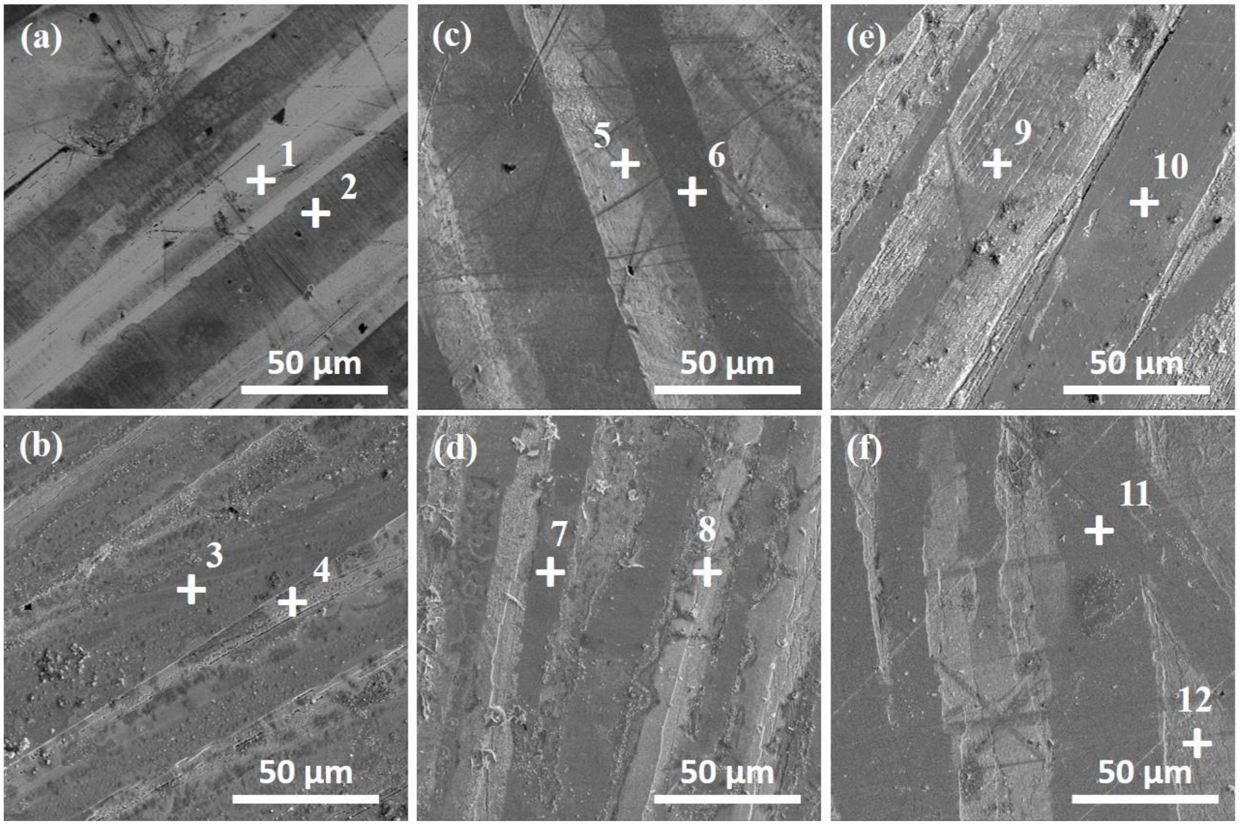

Figure S1. SEM pictures and marked points where the EDS measurements were carried out. (a) $Bi_2Te_{2.7}Se_{0.3}$-A; (b) $Bi_2Te_{2.7}Se_{0.3}$-B; (c) $Bi_2Te_{2.55}Se_{0.45}$-A; (d) $Bi_2Te_{2.55}Se_{0.45}$-B; (e) $Bi_2Te_{2.4}Se_{0.6}$-A; (f) $Bi_2Te_{2.4}Se_{0.6}$-B.

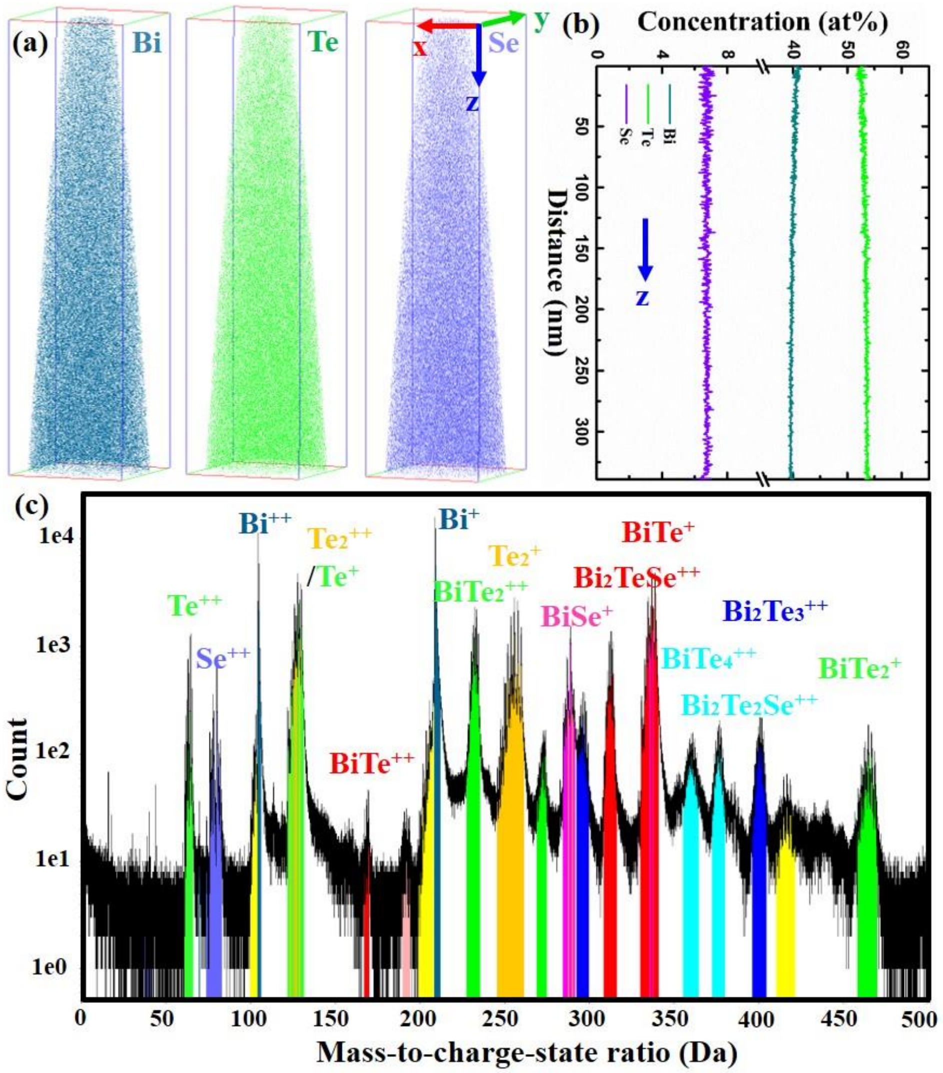

Figure S2. APT results of sample $Bi_2Te_{2.55}Se_{0.45}$-A. (a) Reconstructed three-dimensional distribution of Bi, Te, and Se ions. The volume of the analyzed region is $90 \times 90 \times 340\ nm^3$; (b) 1D concentration profile along the z-axis of the tip using a fixed bin size of 0.5 nm; (c) Mass spectrum of Bi-Te-Se sample showing very complex evaporation process. Only some of the ions are labeled.

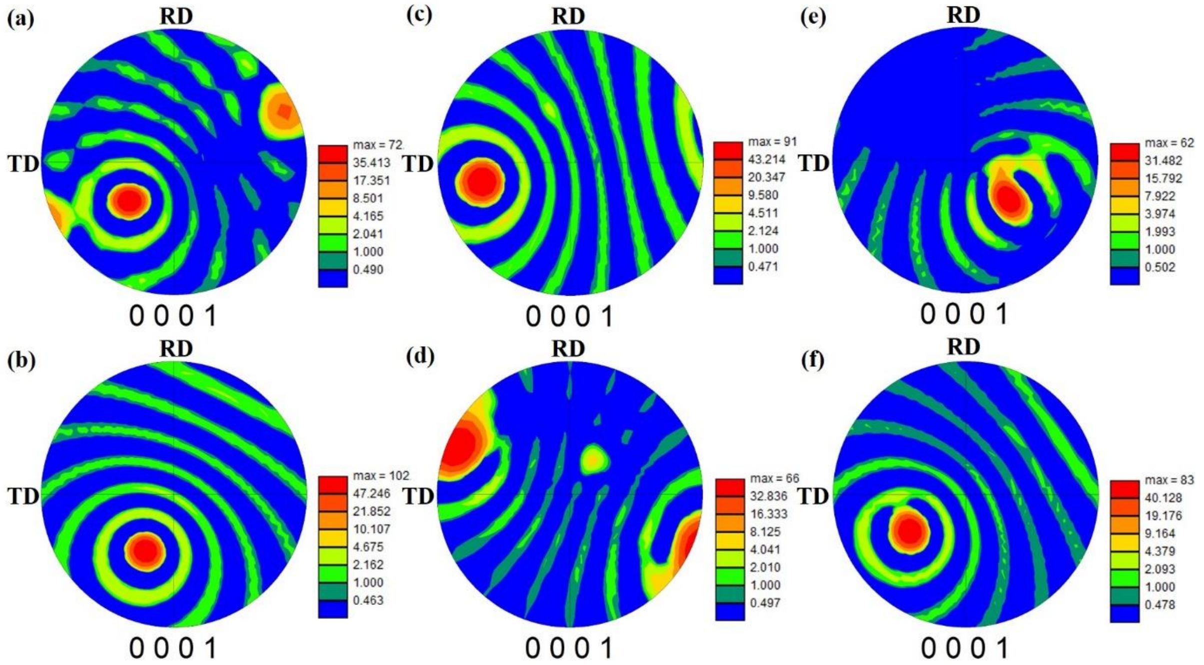

Figure S3. Pole figures of (0001) plane generated by the EBSD resutls as shown in Fig. 7 in the main text. RD and TD represent the rolling and transverse directions, respectively. (a) $Bi_2Te_{2.7}Se_{0.3}$-A; (b) $Bi_2Te_{2.7}Se_{0.3}$-B; (c) $Bi_2Te_{2.55}Se_{0.45}$-A; (d) $Bi_2Te_{2.55}Se_{0.45}$-B; (e) $Bi_2Te_{2.4}Se_{0.6}$-A; (f) $Bi_2Te_{2.4}Se_{0.6}$-B.

Table S1. Characteristic temperatures and parameters in cooling curves of $Bi_2Te_{3-x}Se_x$ melts

Sample	T_N [a] (°C)	T_G [b] (°C)	Δh [c] (°C /s)	t [d] (s)
$Bi_2(Te_{0.9}Se_{0.1})_3$--A	583.9	587.7	6.17	84
$Bi_2(Te_{0.9}Se_{0.1})_3$--B	567.2	582.3	12.55	70
$Bi_2(Te_{0.85}Se_{0.15})_3$--A	589.5	593.2	6.15	84
$Bi_2(Te_{0.85}Se_{0.15})_3$--B	571.9	591.0	13.77	76
$Bi_2(Te_{0.8}Se_{0.2})_3$--A	598.6	604.1	6.89	85
$Bi_2(Te_{0.8}Se_{0.2})_3$--B	584.7	600.4	11.30	73

[a] Starting nucleation and solidification temperature

[b] Crystal growth temperature after recalescence

[c] The height of the first peak for the *dT/dt*-time curve

[d] Crystal growth time

Table S2. Atomic composition ratios of Bi, Te, and Se obtained from the EDS spectra as shown in **Figure S1.** The uncertainty value for EDS is near 3%.

Samples	Spectrum	Bi	Te	Se
x=0.3A	1	39.2	55.2	5.6
	2	39.0	52.1	8.9
x=0.3B	3	37.2	55.6	7.2
	4	37.3	56.1	6.6
x=0.45A	5	36.4	57.5	6.1
	6	40.0	50.5	9.5
x=0.45B	7	38.9	51.4	9.7
	8	34.9	56.0	9.2
x=0.6A	9	37.1	51.2	11.8
	10	38.2	52.5	9.3
x=0.6B	11	39.4	50.8	9.8
	12	37.1	55.1	7.8
